# Microstructural alterations in association tracts and language abilities in schoolchildren born very preterm and with poor fetal growth

**DOI:** 10.1007/s00247-022-05418-3

**Published:** 2022-07-01

**Authors:** Hanna Kallankari, Hanna-Leena Taskila, Minna Heikkinen, Mikko Hallman, Virva Saunavaara, Tuula Kaukola

**Affiliations:** 1 PEDEGO Research Unit and Medical Research Center Oulu, University of Oulu, Oulu, Finland; 2 Department of Child Neurology, Oulu University Hospital, University of Oulu, P.O. Box 5000, FIN-90014 Oulu, Finland; 3 Department of Neonatology, Oulu University Hospital, Oulu, Finland; 4 Child Language Research Center, Faculty of Humanities, University of Oulu, Oulu, Finland; 5 PET Center, Turku University Hospital, Turku, Finland; 6 Department of Medical Physics, Turku University Hospital, Turku, Finland

**Keywords:** Children, Diffusion tensor imaging, Language outcome, Magnetic resonance imaging, Preterm, Reading ability, White matter tracts

## Abstract

**Background:**

Prematurity and perinatal risk factors may influence white matter microstructure. In turn, these maturational changes may influence language development in this high-risk population of children.

**Objective:**

To evaluate differences in the microstructure of association tracts between preterm and term children and between preterm children with appropriate growth and those with fetal growth restriction and to study whether the diffusion tensor metrics of these tracts correlate with language abilities in schoolchildren with no severe neurological impairment.

**Materials and methods:**

This study prospectively followed 56 very preterm children (mean gestational age: 28.7 weeks) and 21 age- and gender-matched term children who underwent diffusion tensor imaging at a mean age of 9 years. We used automated probabilistic tractography and measured fractional anisotropy in seven bilateral association tracts known to belong to the white matter language network. Both groups participated in language assessment using five standardised tests at the same age.

**Results:**

Preterm children had lower fractional anisotropy in the right superior longitudinal fasciculus 1 compared to term children (*P* < 0.05). Preterm children with fetal growth restriction had lower fractional anisotropy in the left inferior longitudinal fasciculus compared to preterm children with appropriate fetal growth (*P* < 0.05). Fractional anisotropy in three dorsal tracts and in two dorsal and one ventral tract had a positive correlation with language assessments among preterm children and preterm children with fetal growth restriction, respectively (*P* < 0.05).

**Conclusion:**

There were some microstructural differences in language-related tracts between preterm and term children and between preterm children with appropriate and those with restricted fetal growth. Children with better language abilities had a higher fractional anisotropy in distinct white matter tracts.

## Introduction

Although the survival of infants born before 32 weeks of gestation has improved in the past 20 years, long-term neurocognitive outcomes remain a major concern [1]. Neurodevelopmental deficits in these children, such as specific language problems, have been linked to compromised educational performance [2] that often persists from childhood into adolescence and young adulthood [3]. After preterm birth, white matter is susceptible to injuries that affect neuronal development [4]. Aside from preterm birth, a range of perinatal factors, including fetal growth restriction and neonatal diseases may influence white matter maturation [5–7]. Specific magnetic resonance imaging (MRI) sequences, including diffusion tensor imaging and advanced neuroinformatic techniques such as probabilistic tractography, offer an opportunity to study white matter microstructures [8] and to correlate the findings with prenatal and postnatal complications, as well as later emerging developmental impairments. Quantitative measures of water diffusion in white matter, particularly fractional anisotropy, reflect the organisation of neural pathways and have been found to relate to language abilities in children with atypical and typical language development [9] and in preterm children in some [10] but not all studies [11]. There are also limited data concerning the relationship between microstructural findings in white matter and language development in very preterm children with poor fetal growth.

As part of our prospective cohort study, children born at a very low gestational age (VLGA) and a comparison group of term children underwent diffusion tension imaging and language assessment at 9 years of age. The follow-up age of 9 years was chosen because children have usually achieved good language comprehension, naming and technical reading skills by then. In addition, although still maturing, the white matter of children at school age has a structural and functional connectivity more closely resembling that of adults compared to infants or preschool children [12, 13]. We have previously shown that the VLGA children had poorer performance in reading comprehension and spelling at 9 years of age [14].

In this study, we defined seven association white matter tracts previously shown to be related to language development using automated probabilistic tractography and quantitated diffusion tensor imaging metrics. Our aim was to test the hypotheses that fractional anisotropy values in these tracts would be different between preterm and term children and between preterm children with and without fetal growth restriction. Our further aim was to study whether fractional anisotropy values would be associated with language skills in these children.

## Materials and methods

### Subjects

VLGA children enrolled in the present study belonged to a prospectively collected cohort of children born before 32 weeks of gestation at Oulu University Hospital between November 1998 and November 2002. Serial brain ultrasound (US) examinations were performed during the neonatal period. Severe brain injury was defined as intraventricular haemorrhage grade 3 or 4 [15] or cystic periventricular leukomalacia [16]. Other characteristics of this population, including fetal growth restriction, have previously been described in detail [6, 17]. A group of age- and gender-matched term children was selected from the birth register of Oulu University Hospital. The recruitment and follow-up assessments of both the VLGA and term children were carried out during a four-year period, between November 2007 and November 2011, at Oulu University Hospital [6, 14]. The study was approved by the Ethics Committee of Oulu University Hospital, and written informed content was obtained from both the participating children and their parents.

Among the VLGA children, the diagnosis of cerebral palsy was set by a child neurologist and confirmed at the age of 5 years based on criteria established by the Surveillance of Cerebral Palsy in Europe network. Every child who participated in the present study also underwent a structured neurological assessment at the age of 9 years. None of the term children was diagnosed with cerebral palsy. VLGA children who had severe cognitive impairment, an intelligence quotient < 50, were blind or did not speak Finnish were excluded from the follow-up [14]. All participants attended mainstream schools, including three VLGA children who had received a diagnosis of mild intellectual disability on a clinical basis before entry into the study.

Sixty-eight VLGA and 23 term children underwent brain MRI at a mean age of 9 years (range: 8.6–9.6 years). Twelve VLGA children were excluded from the diffusion tensor imaging study. The exclusion criteria were cerebral palsy (*n* = 4), missing diffusion tensor imaging data (*n* = 1), and problems with MRI data transfer (*n* = 2) or with quality control criteria (*n* = 5). Two term children were excluded because of missing diffusion tensor imaging data. Five VLGA children and one term child did not participate in language assessments and three VLGA children were further excluded because of mild intellectual disability. Thus, the final study group included 48 VLGA and 20 term children with both diffusion tensor imaging data and language assessments.

### Language assessments

A speech therapist with 26 years of experience (M.H.) performed the language assessments using standardised tests. The Token test measures the comprehension of verbal instructions of increasing complexity [18], the Rapid naming test measures the ability to recognise a visual symbol such as a letter or a number and to name it accurately and rapidly [19, 20] and the Word chain test evaluates word reading skills (word recognition, identification between real words and pseudo words and word segmentation) [21]. To reduce the number of outcome variables, we calculated composite scores for each test to be used in analyses. Two tests for Finnish-speaking primary school–aged children (YTTE, measuring reading fluency and comprehension, and Lukilasse, assessing spelling), previously described in detail [14], were further used to evaluate reading skills.

### Neuroimaging

Magnetic resonance imaging data were collected using a 1.5-T GE Signa HDX MRI scanner (General Electrics, Milwaukee, WI) at Oulu University Hospital. An 8-channel high resolution brain coil was used. The child’s head was surrounded by soft cushions during scanning and earplugs were used to protect the child from imaging noise. According to the protocol no sedation was used during imaging.

The study protocol included a T1-weighted sagittal spin echo. For this protocol, the slice thickness was 5 mm with a 1-mm gap between slices, the field of view was 24 cm with a 512 × 512 matrix, the repetition time was 540 ms and the echo time was 14 ms. In addition, T2-weighted axial images were taken using the PROPELLER (periodically rotated overlapping parallel lines with enhanced reconstruction) technique with a slice thickness of 5 mm and a 1-mm gap between slices, an echo train length of 28, a reconstruction diameter of 22 cm with a 512 × 512 matrix, a repetition time of 5,000 ms and an echo time of 172.592 ms. Diffusion-weighted images (DWI) were acquired using a spin echo echo planar sequence with an isotropic 3-mm voxel, 40 directions and a b value of 1,000. The repetition time was 9,000 ms and the echo time was as short as possible. The slice thickness was 3 mm and the field of view was 19.2 cm with a 64 × 64 matrix.

The diffusion tensor imaging analyses were interpreted by a medical physicist (V.S.) with 7 years of experience in medical imaging. Data quality control was carried out with DTIPrep (Universities of North Carolina, Iowa and Utah, USA) [22]. Data were checked for slice-wise and interlace-wise intensity differences. Eddy current and motion defects were corrected, and data were checked gradient-wise for residual motion or deformations. Gradient directions that had image artefacts were removed from the data.

**Fig. 1:**
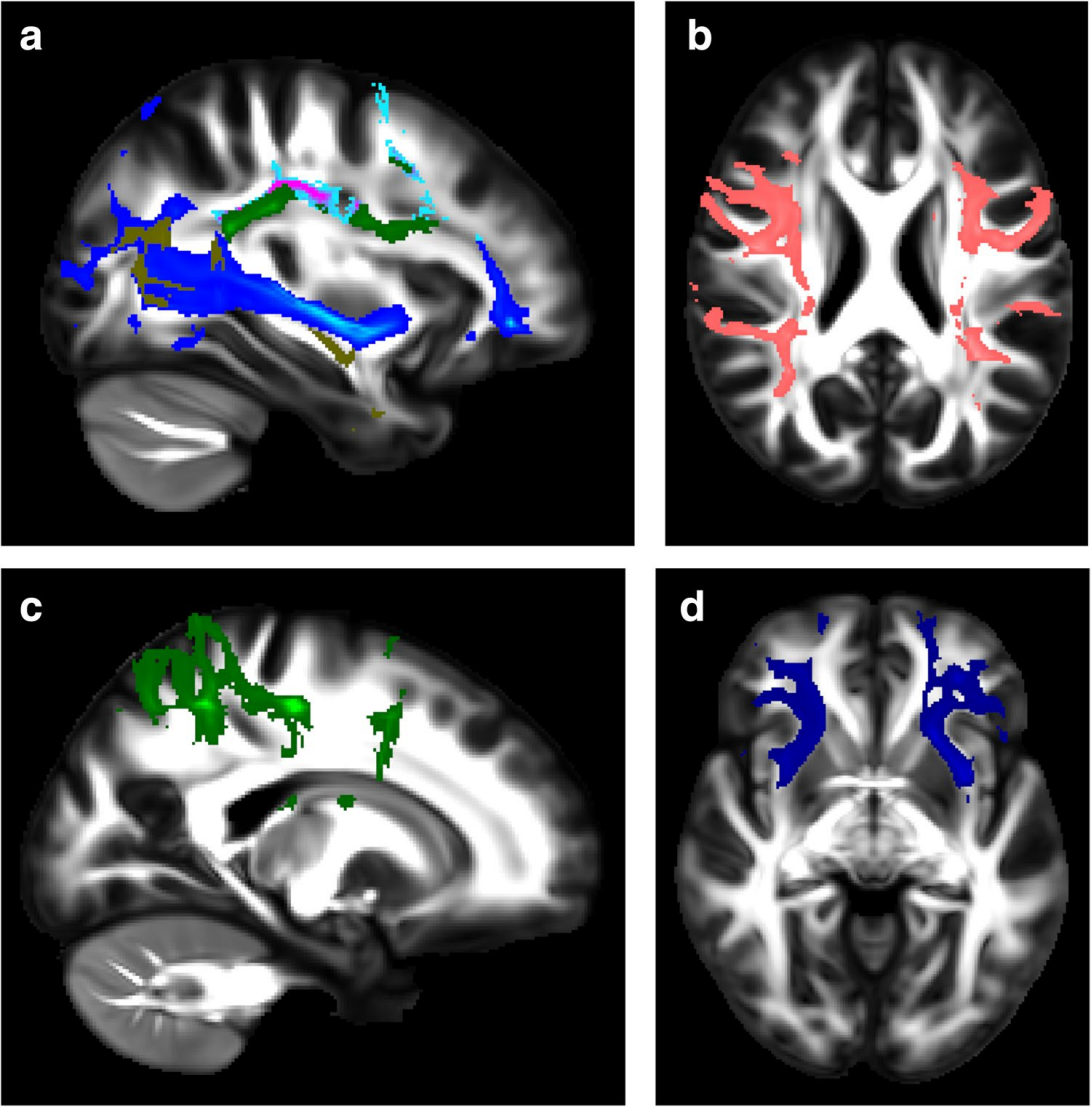
T1-weighted images with an overlay of the language-related tracts coded by colours in a 9.1-year-old healthy control girl who had normal findings on conventional magnetic resonance imaging. **a **A left sagittal image of the superior longitudinal fasciculus 2 is displayed in turquoise/pink, the superior longitudinal fasciculus 3 in green, the inferior fronto-occipital fasciculus in blue/light blue and the inferior longitudinal fasciculus in dark yellow. **b **An axial image with the left and right arcuate fasciculi displayed in red. **c **A left sagittal image with the superior longitudinal fasciculus 1 displayed in green. **d **An axial image with the left and right uncinate fasciculi displayed in blue

Data preparations for tractography analysis were carried out using the Functional Magnetic Resonance Imaging of the Brain (FMRIB) Software Library (FSL) version 5.011 (FMRIB Analysis Group, Oxford, UK) and XTRACT analysis with FSL version 6.0.4 [23]. Brain was extracted using FSL’s Brain Extraction Tool [24], diffusion metrics were calculated and a crossing fibre model was fitted using FMRIB’s Diffusion Toolbox [25, 26]. Fractional anisotropy images were registered to structural T1-weighted data and then to standard Montréal Neurological Institute (MNI) 152 space using FMRIB’s Linear Image Registration tool [27]. Warp fields were obtained using FSL’s FNIRT tool [28]. Probabilistic tractography was carried out using XTRACT, which is a new software tool with a library of standardised tractography protocols devised for the robust automated extraction of white matter tracts [29]. The XTRACT stats tool was used to extract tract summary statistics (including fractional anisotropy) from the studied tracts: the arcuate fasciculus, three branches of the superior longitudinal fasciculus, inferior longitudinal fasciculus, inferior fronto-occipital fasciculus, and uncinate fasciculus (Fig. 1). Data were visualised with FSLeyes utilising the XTRACT_viewer.

### Statistical analyses

**Fig. 2:**
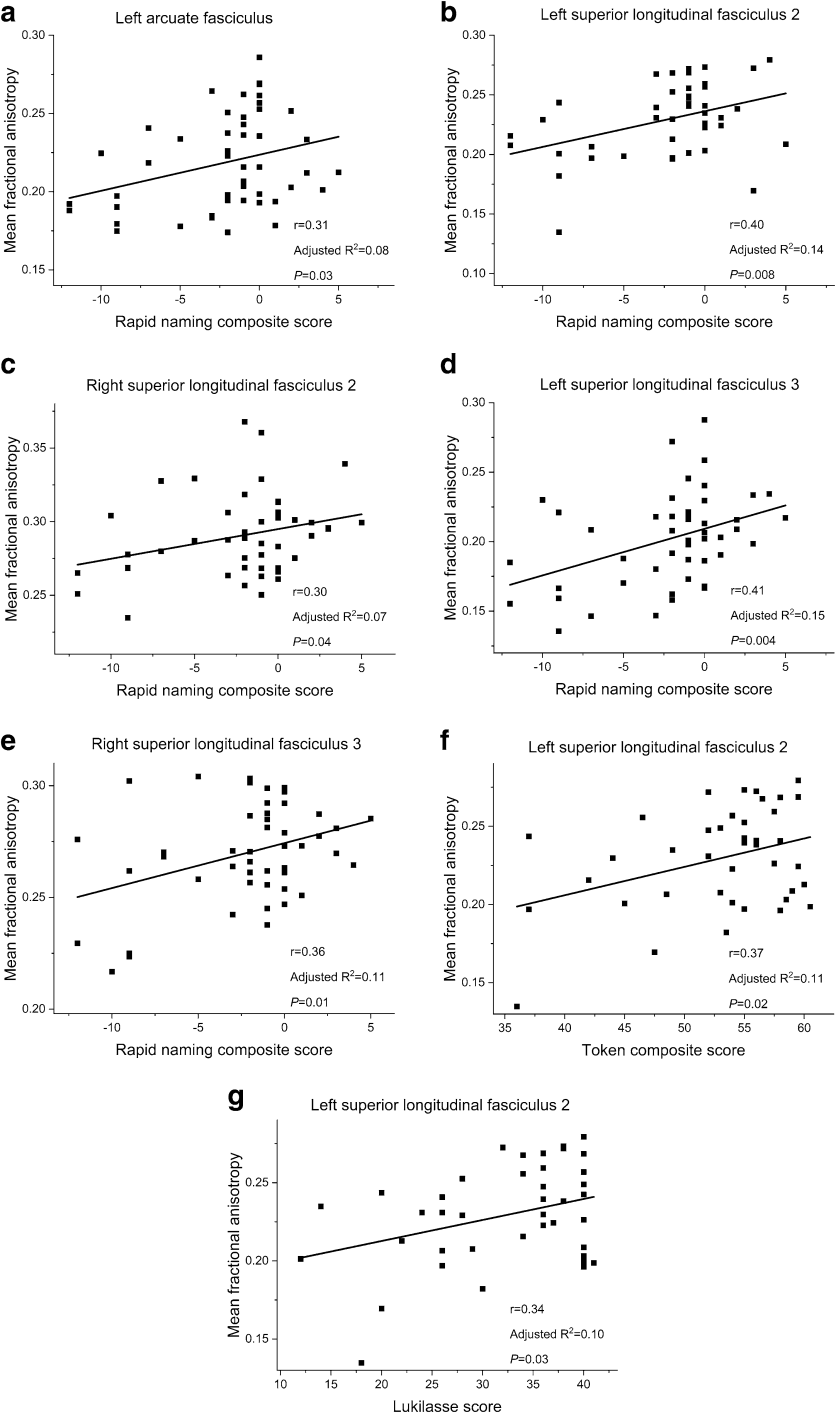
Graphs (**a**−**g**) show the correlation between language testing scores on the *x*-axis and mean fractional anisotropy values in specific tracts on the *y*-axis, which were significant in the univariate linear regression analyses for very low gestational age children. Results remained significant after controlling for gestational age, gender, perinatal brain injury and maternal education level. Each black square represents data from a single subject

**Fig. 3:**
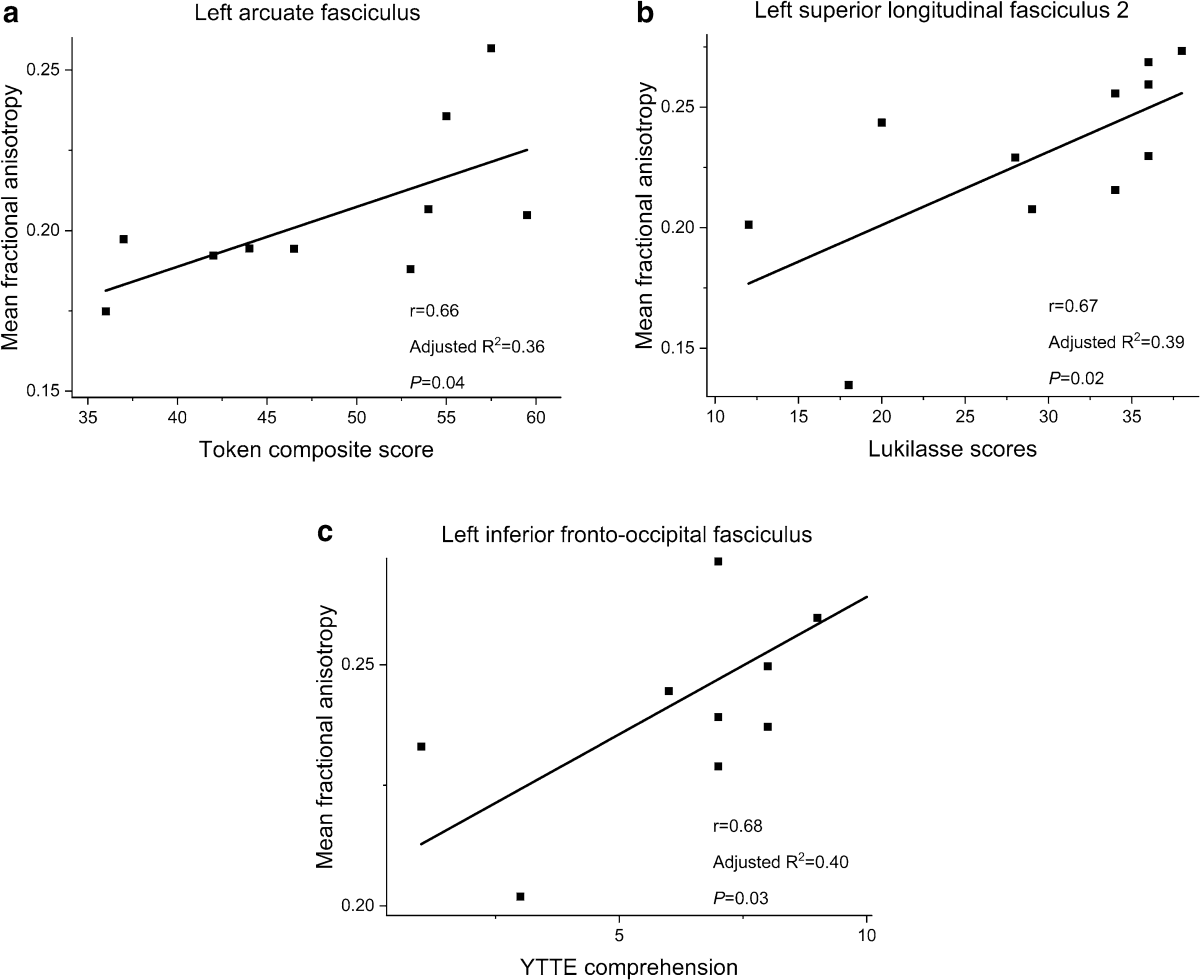
Graphs (**a**−**c**) show the correlation between language testing scores on the *x-*axis and mean fractional anisotropy values in specific tracts on the *y-*axis, which were significant in the univariate linear regression analyses for very low gestational age children with fetal growth restriction. Results remained significant after controlling for gestational age, gender and maternal education level. Each black square represents data from a single subject

Statistical analyses were performed using SPSS 26.0 (SPSS Inc., Chicago, IL). The differences in the averaged fractional anisotropy values in each tract and on both sides between the two groups were evaluated using Student’s *t*-tests. Due to our sample size, the number of confounding variables that could be included in the analyses was limited. The following pre- and postnatal factors-fetal growth restriction, histological chorioamnionitis, antenatal steroids, gender, gestational age at birth, respiratory distress syndrome, severe perinatal brain injury, bronchopulmonary dysplasia and maternal educational level-were correlated with the fractional anisotropy values and with the language scores. Factors that correlated significantly with the outcome variables were then included in the analyses as confounding variables. Linear regression models with confounding variables were conducted to control the *t*-test results and to evaluate correlations between the fractional anisotropy values and language scores (Figs. 2 and 3). The level of significance was set at *P* < 0.05, two-tailed.

## Results

Table 1 shows the clinical characteristics of the participants. Four VLGA children had severe perinatal brain injury. The VLGA children were found to have significantly lower fractional anisotropy in the right superior longitudinal fasciculus 1 than the term children (Table 2). The VLGA children with poor fetal growth had lower fractional anisotropy in the left inferior longitudinal fasciculus than the VLGA children with appropriate fetal growth (Table 3).

**Table 1:** Clinical characteristics of the study population

	VLGA children (*n* = 56)	Term children (*n* = 21)
Boys, *n* (%)	30 (54)	11 (52)
Gestational age, mean in weeks (range)	28.7 (24.1–31.9)	39.4 (37.3–41.6)
Birth weight, mean in grams (range)	1,162 (495–2,295)	3,356 (2,655–4,040)
Fetal growth restriction, *n* (%)	13 (23)	NA
Antenatal steroids, *n* (%)	48 (86)	NA
Intraventricular haemorrhage grade 2, *n* (%)	3 (5.4)	NA
Intraventricular haemorrhage grade 3, *n* (%)^a^	3 (5.4)	NA
Periventricular leukomalacia, grade 1, *n* (%)	12 (21)	NA
Cystic periventricular leukomalacia, *n* (%)	1 (1.8)	NA
High maternal education level, *n* (%)^b^	31 (57)	11 (55)
Speech therapy^c^	19 (36)	3 (15)
Age at the DTI scanning, mean in years (range)	9.0 (8.6–9.6)	9.1 (8.8–9.3)

*DTI *Diffusion tensor imaging,* NA* not available, *VLGA* very low gestational age

^a^ None of the VLGA children had intraventricular haemorrhage grade 4

^b^ High education level refers to a 12-year education of comprehensive and upper-secondary school plus vocational education, polytechnic or university degree. Data are missing for two VLGA children and one term child

^c^ Data are missing for three VLGA children and one term child

**Table 2:** Comparison of fractional anisotropy of the association tracts between 56 very low gestational age (VLGA) and 21 term-born children

Tract	VLGA^a^	Term^a^	*P-*value^b^
Arcuate fasciculus, left	0.219 (0.028)	0.218 (0.022)	0.94
Arcuate fasciculus, right	0.305 (0.022)	0.301 (0.022)	0.48
Superior longitudinal fasciculus 1, left	0.286 (0.043)	0.289 (0.024)	0.69
Superior longitudinal fasciculus 1, right	0.215 (0.045)	0.239 (0.036)	0.03^c^
Superior longitudinal fasciculus 2, left	0.230 (0.030)	0.226 (0.031)	0.67
Superior longitudinal fasciculus 2, right	0.293 (0.028)	0.296 (0.028)	0.63
Superior longitudinal fasciculus 3, left	0.202 (0.032)	0.215 (0.031)	0.12
Superior longitudinal fasciculus 3, right	0.269 (0.023)	0.257 (0.028)	0.06
Uncinate fasciculus, left	0.214 (0.029)	0.201 (0.019)	0.05
Uncinate fasciculus, right	0.229 (0.025)	0.223 (0.020)	0.34
Inferior longitudinal fasciculus, left	0.232 (0.038)	0.231 (0.031)	0.95
Inferior longitudinal fasciculus, right	0.235 (0.026)	0.238 (0.025)	0.74
Inferior fronto-occipital fasciculus, left	0.255 (0.028)	0.251 (0.023)	0.60
Inferior fronto-occipital fasciculus, right	0.254 (0.024)	0.249 (0.022)	0.35

^a^ Data are given as mean (standard deviation)

^b^ Student’s *t*-test

^c^ This result remained significant after controlling for gender and perinatal brain injury

**Table 3:** Comparison of fractional anisotropy of the association tracts between 13 very low gestational age (VLGA) children with fetal growth restriction (FGR) and 43 appropriately grown VLGA children

Tract	FGR^a^	AGA^a^	*P*-value^b^
Arcuate fasciculus, left	0.207 (0.022)	0.222 (0.029)	0.09
Arcuate fasciculus, right	0.298 (0.025)	0.307 (0.020)	0.20
Superior longitudinal fasciculus 1, left	0.284 (0.032)	0.287 (0.046)	0.83
Superior longitudinal fasciculus 1, right	0.210 (0.048)	0.216 (0.044)	0.66
Superior longitudinal fasciculus 2, left	0.228 (0.038)	0.230 (0.027)	0.83
Superior longitudinal fasciculus 2, right	0.280 (0.025)	0.297 (0.028)	0.06
Superior longitudinal fasciculus 3, left	0.193 (0.027)	0.204 (0.034)	0.26
Superior longitudinal fasciculus 3, right	0.261 (0.029)	0.272 (0.021)	0.16
Uncinate fasciculus, left	0.208 (0.024)	0.216 (0.030)	0.37
Uncinate fasciculus, right	0.231 (0.023)	0.228 (0.026)	0.74
Inferior longitudinal fasciculus, left	0.211 (0.026)	0.238 (0.040)	0.02^c^
Inferior longitudinal fasciculus, right	0.231 (0.030)	0.237 (0.025)	0.48
Inferior fronto-occipital fasciculus, left	0.242 (0.020)	0.259 (0.030)	0.07
Inferior fronto-occipital fasciculus, right	0.260 (0.017)	0.252 (0.025)	0.32

*AGA* appropriate for gestational age

^a^ Data are given as mean (standard deviation)

^b^ Student’s *t*-test

^c^ This result remained significant after controlling for gestational age, gender and perinatal brain injury

In the VLGA children, low fractional anisotropy in the left arcuate fasciculus, the left and right superior longitudinal fasciculus 2, and the left and right superior longitudinal fasciculus 3 correlated significantly with low Rapid naming scores (Fig. 2). After adding the abovementioned five mean fractional anisotropy values to the linear regression model, fractional anisotropy in the left superior longitudinal fasciculus 3 was found to be the most significant predictor of Rapid naming scores (r = 0.418, *P* = 0.007). In addition, low fractional anisotropy in the left superior longitudinal fasciculus 2 correlated significantly with low Token scores and with low Lukilasse scores (Fig. 2). Among the VLGA children with fetal growth restriction, low fractional anisotropy in the left arcuate fasciculus correlated significantly with low Token scores. Low fractional anisotropy in the left superior longitudinal fasciculus 2 correlated with low Lukilasse scores and low fractional anisotropy in the left inferior fronto-occipital fasciculus correlated with low YTTE comprehension scores (Fig. 3). In the term children, low fractional anisotropy in the left superior longitudinal fasciculus 1 correlated significantly with low Rapid naming scores (r = 0.486, *P* = 0.035). Otherwise, fractional anisotropy values were not associated significantly with language scores in term children (data not shown).

## Discussion

This study showed differences in diffusion tensor imaging metrics in the right superior longitudinal fasciculus 1 between the VLGA children with no severe neurological impairment and term children at 9 years of age. The VLGA children with fetal growth restriction had differences in the left inferior longitudinal fasciculus compared to the VLGA children with appropriate fetal growth. Furthermore, the VLGA children and the subgroup of preterm children with fetal growth restriction demonstrated a distinct pattern of correlation between diffusion tensor imaging metrics and language skills at school age.

Good language abilities are crucial to academic and occupational success [9]. Such skills usually improve with age as the brain matures and are supported by a wide language network involving association tracts that connect different areas in the same hemisphere. The dorsal language stream, including arcuate fasciculus and three branches of the superior longitudinal fasciculus, links the frontal and temporoparietal areas and is suggested to be involved in the auditory-to-motor mapping of speech sounds and articulation, phonological processing, repetition and processing of complex sentences. The ventral stream, including the inferior longitudinal fasciculus, inferior fronto-occipital fasciculus and uncinate fasciculus, connects the frontal, occipital and temporal regions and has been related to the ability to understand the meanings of words and sentences, as well as the visual aspect of reading [11, 30].

We found that VLGA children had significantly lower fractional anisotropy values in the right superior longitudinal fasciculus 1 than term children. Supporting our findings, other studies have also found lower fractional anisotropy values in preterm children compared to those born at term in the abovementioned tract [31]. However, there are studies that show only limited or no differences [11, 32]. In general, fractional anisotropy indicates restricted water movement across the axonal fibre, and values tend to increase consistent with white matter maturation, possibly indicating increased myelination and more coherent axonal structures [31]. We did not find differences in fractional anisotropy values in any other white matter tracts between the VLGA and term groups. This might be because we included only those who had no major neurological impairments, and only 6 VLGA children were born before 26 weeks of gestation. As perinatal and neonatal care has advanced, less severe medical problems are seen in preterm infants, which may also have propitious effects on white matter development [7, 32]. In addition, groups may differ in scanning age, perinatal risk factors or environmental factors that operate beyond the neonatal period. These may all contribute to differing results between studies.

We further showed that the VLGA children with fetal growth restriction had lower fractional anisotropy values in the left inferior longitudinal fasciculus compared to VLGA children with appropriate fetal growth. Two previous studies have demonstrated fractional anisotropy alterations in multiple white matter tracts, including the inferior longitudinal fasciculus, in preterm infants with poor fetal growth [7, 33]. In our study, the number of children with poor fetal growth was small, and none of them had suffered from intraventricular haemorrhage grade 3−4 or cystic periventricular leukomalacia. This might explain why only minor differences were found between the VLGA children with fetal growth restriction and those with appropriate growth in the current study.

Little is known about the correlations between white matter microstructures and different components of language among preterm children [10]. We observed that fractional anisotropy values in the left arcuate fasciculus and bilaterally in the superior longitudinal fasciculi 2 and 3 were positively associated with rapid naming — fractional anisotropy in the left superior longitudinal fasciculus 3 being the most significant predictor of this phonological ability. Furthermore, fractional anisotropy in the left superior longitudinal fasciculus 2 was positively associated with the comprehension of verbal instructions and spelling. In addition to our study, previous studies have also found correlations between fractional anisotropy in dorsal routes and language abilities in preterm children and adolescents [34–37]. In line with our findings, Mullen et al. [34] showed that phonological tasks were positively correlated with fractional anisotropy in the arcuate fasciculus, but bilaterally. Furthermore, Travis et al. [35] demonstrated that reading skills were positively associated with fractional anisotropy in the arcuate fasciculus and in segments of anterior superior longitudinal fasciculus. In contrast, Bruckert et al. [11] found that fractional anisotropy in dorsal language pathways was related to reading outcomes in term but not preterm children. In our study, term children with high fractional anisotropy in the left superior longitudinal fasciculus 1 had better naming skills. The correlation variability between fractional anisotropy and language functions in different studies may be due to differences in MRI and neuroinformatic methodology (e.g., extraction of the tracts), tests measuring language functioning or the developmental stages of specific white matter areas [31].

In the present study, fractional anisotropy in two dorsal tracts and in one ventral tract showed correlations with language abilities in VLGA children with fetal growth restriction. Fractional anisotropy in the left arcuate fasciculus had a positive correlation with verbal comprehension. Fractional anisotropy in the left superior longitudinal fasciculus 2 and in the left inferior fronto-occipital fasciculus were positively correlated with reading abilities. To our knowledge, few studies have reported associations between diffusion indices and language outcomes among very preterm children with fetal growth restriction. However, poor fetal growth has been found to affect a wide range of neurodevelopmental abilities, including language skills [5, 38]. We have previously shown that poor fetal growth is a risk factor for reading difficulties at school age [14].

The current study protocol included brain US to diagnose periventricular leukomalacia and intraventricular haemorrhage during the neonatal period. Although US has limited value to detect diffuse white matter injury, it has been shown to have good reliability in detecting cystic periventricular lesions, intraventricular haemorrhages and haemorrhagic parenchymal infarctions compared to MRI [39, 40]. In our study, four VLGA children had severe brain injury during the neonatal period. However, this was not found to affect the correlation between language skills and fractional anisotropy in specific white matter tracts.

The present study provides insights into the neural basis of language skills between VLGA children (considering those with fetal growth restriction as a subgroup) and term children at school age. Interestingly, low scores in the language assessments correlated with low fractional anisotropy mainly in the left-side tracts among preterm children and preterm children with fetal growth restriction. This finding may reflect altered brain lateralisation reported among prematurely born children and adolescents [41]. However, interpretation of the exact relationship between diffusion tensor imaging measures, in particular fibre route and language abilities remains to be articulated, as language functions are driven by a combination of complex pathways involving several brain areas [10]. Recently, it has also been shown that microstructural development is highly variable even among individual preterm infants at term age [42].

The strengths of this study relate to our study design. Our VLGA population was well defined and prospectively recruited [6, 17], and the comparison group of term children was an age- and gender-matched representative sample of 9-year-old schoolchildren with no severe neurological impairments. All participants underwent objective language evaluations, including both expressive and perceptive language abilities, as well as reading skills and diffusion tensor imaging examining both the ventral and dorsal association pathways within the same age range. The same MRI scanner was used to obtain diffusion tensor imaging sequences for all children in the study. The diffusion tensor imaging analyses were performed using well-defined techniques, of which the user independent XTRACT method was a special strength [29]. Imaging techniques such as simultaneous multi-slice excitation or increased number of directions might have improved data quality. However, we only had quality issues with five children, who were excluded from the analyses. We could have used higher level models in the analyses if the data were acquired using higher number of directions and b values. Nevertheless, it is uncertain if either of these changes would have impacted the results of this study. A possible limitation of our study was the small group size of term children and children with fetal growth restriction, which could have affected hypothesis testing. Due to our sample size, although it was in line with previous diffusion tensor imaging studies [10, 31], we chose not to correct for multiple comparisons to avoid potential type II errors. The comparisons are not independent, either—the language outcomes are dependent on one another as are the MRI parameters. The results should therefore be interpreted with caution [43]. Also, a possibility of margin of error in language testing should be considered when interpreting the outcomes of this study.

In the future, interest will shift from single brain regions to a comprehensive mapping of whole brain connectivity. Combining both functional and structural neuroimaging using novel techniques that allow better identification of multiple crossing fibres, for example, and having larger cohorts with longitudinal follow-up may increase our understanding of the long-term neurodevelopmental sequelae of very preterm birth [44].

## Conclusion

We used well-defined diffusion metrics to study microstructural findings in seven association tracts known to belong to the language network. We demonstrated that preterm children had a lower fractional anisotropy in one unilateral dorsal language tract compared to term children at 9 years of age. Preterm children with fetal growth restriction had a lower fractional anisotropy in one unilateral ventral language tract compared to those with appropriate growth. The results of well-standardised language tests, evaluating both expressive and perceptive language, showed positive correlation with fractional anisotropy in six association tracts altogether. The preterm children had more microstructural findings in the white matter that were related to language skills compared to the term children. This may indicate altered white matter maturation, which appears even at 9 years of age among these children. Our findings underline the importance of early detection and management of language difficulties in children born very preterm.
